# 

**Published:** 1852-07

**Authors:** 


*New Publications.*—Our table is loaded with new books, and various publications requiring editorial attention. Absence from our post for a couple of weeks, and pressing duties since our return, have prevented us from bestowing upon them that examination which they claim. Our next issue will probably afford evidence of progress in the discharge of this duty. We beg those interested to accept our apology for an apparent remissness in this matter.

---

*Notice.* — G. H. Derby & Co. will publish, for the author, on or about the 1st of August, a volume, entitled "Clinical Reports on Continued Fever, based on analyses of one hundred and sixty-four cases; with remarks on the management of Continued fever, the identity of Typhus and Typhoid fever, Relapsing fever, etc. To which is added, a memoir on the transportation and diffusion, by contagion, of Typhoid fever, as exemplified in the occurrence of the disease at North Boston, Erie county, N. Y. By Austin Flint, M. D., Professor of the Principles and Practice of Medicine, and of Clinical Medicine, in the University of Buffalo, and Editor of the Buffalo Medical Journal." The volume will consist, mainly, of Reports, etc., that have appeared in the Buffalo Medical Journal, volumes V. VI. and VII. It will form an octavo of about four hundred pages, and will be furnished, neatly bound in cloth, at $2. Copies will be sent, with the postage prepaid, through the post-office, to persons remitting two dollars, provided the distance does not exceed five hundred miles. Orders may be addressed to G. H. Derby & Co., or to the Editor of the Buffalo Medical Journal.

---

*Lost Letter.* — Some time in May a letter, post marked Montreal, addressed to the editor of this Journal, was lost by a messenger bringing letters from the post office, and has not been recovered. This notice is made under the probability that it related to the Journal, and the fact of the loss will, in that case, reach the eye of the unknown correspondent.

---

*Missing Communication.* — About the middle of May last, we received a communication for this Journal, which we did not examine, being about to leave town for an absence of a fortnight. On returning we were unable to find the communication referred to. Will the author, whose name and residence have escaped us, favor us with a line, or, if practicable, send a duplicate?

---

A communication from Prof. Hamilton received too late for insertion in the present No., will appear in our next.

A highly interesting letter by Prof. Dalton, written at Paris, will also receive insertion in the No. for August.

---

*New Haven Com. Medical College.* — Dr. Worthington Hooker, author of "Physician and Patient," and other able and popular works, has recently received the appointment of Professor of the Theory and Practice of Medicine in the Medical department of Yale University.

